# Functional Bioassays for Immune Monitoring of High-Risk Neuroblastoma Patients Treated with ch14.18/CHO Anti-GD_2_ Antibody

**DOI:** 10.1371/journal.pone.0107692

**Published:** 2014-09-16

**Authors:** Nikolai Siebert, Diana Seidel, Christin Eger, Madlen Jüttner, Holger N. Lode

**Affiliations:** Department of Pediatric Oncology and Hematology, University Medicine Greifswald, Greifswald, Germany; Gustave Roussy, France

## Abstract

Effective treatment of high-risk neuroblastoma (NB) remains a major challenge in pediatric oncology. Human/mouse chimeric monoclonal anti-GD_2_ antibody (mAb) ch14.18 is emerging as a treatment option to improve outcome. After establishing a production process in Chinese hamster ovary (CHO) cells, ch14.18/CHO was made available in Europe for clinical trials. Here, we describe validated functional bioassays for the purpose of immune monitoring of these trials and demonstrate GD_2_-specific immune effector functions of ch14.18/CHO in treated patients. Two calcein-based bioassays for complement-dependent- (CDC) and antibody-dependent cellular cytotoxicity (ADCC) were set up based on patient serum and immune cells tested against NB cells. For this purpose, we identified LA-N-1 NB cells as best suited within a panel of cell lines. Assay conditions were first established using serum and cells of healthy donors. We found an effector-to-target (E:T) cell ratio of 20∶1 for PBMC preparations as best suited for GD_2_-specific ADCC analysis. A simplified method of effector cell preparation by lysis of erythrocytes was evaluated revealing equivalent results at an E:T ratio of 40∶1. Optimal results for CDC were found with a serum dilution at 1∶8. For validation, both within-assay and inter-assay precision were determined and coefficients of variation (CV) were below 20%. Sample quality following storage at room temperature (RT) showed that sodium-heparin-anticoagulated blood and serum are stable for 48 h and 96 h, respectively. Application of these bioassays to blood samples of three selected high-risk NB patients treated with ch14.18/CHO (100 mg/m^2^) revealed GD_2_-specific increases in CDC (4.5–9.4 fold) and ADCC (4.6–6.0 fold) on day 8 compared to baseline, indicating assay applicability for the monitoring of multicenter clinical trials requiring sample shipment at RT for central lab analysis.

## Introduction

Monoclonal antibodies targeting disialoganglioside GD_2_ emerge as an important treatment option for NB, a dismal pediatric malignancy characterized by high expression of GD_2_ on tumor cells [1], [2]. Ganglioside GD_2_ is a glycolipid antigen devoid of an intracellular signal transduction domain. Therefore the mechanism of action of anti-GD_2_ monoclonal Ab mostly rely on immune effector functions mediated by mAbs, which are more and more recognized as the key features of this class of cancer therapeutics [3]. These features include the activation of CDC and ADCC.

CDC is induced through binding of a serine protease complex C1 to the Fc domains of two or more mAbs binding to antigens expressed on tumor cells. This classical complement pathway results in an activation cascade resulting in the membrane attack complex disrupting the target cell. ADCC is a result of Fc-gamma receptor (FcγR) mediated interaction with effector immune cells such as natural killer (NK) cells, macrophages and granulocytes [3]. The binding of FcγR to Fc domain induces both release of granzymes and perforin from effector cells leading to a target cell lysis and Fc-dependent tumor cell phagocytosis.

The clinical development of anti-GD_2_ monoclonal antibodies for NB patients originated from the discovery of two distinct murine anti-GD_2_ antibodies designated 3F8 [4] and 14.18 [5], respectively. High-risk NB patients were successfully treated within clinical trials with both antibodies mostly conducted by cooperating academic groups of pediatric oncologists. In a more multi center and international approach, the human/mouse chimeric version of 14.18 (ch14.18) has demonstrated activity and efficacy as a monotherapy [6], [7] and in combination with cytokines [8]. In Europe, ch14.18 antibody was made available for clinical trials following the recloning of the antibody genes into CHO cells which was designated as ch14.18/CHO. This is important, as ch14.18/CHO revealed superior activity in mediating ADCC compared to ch14.18 antibody produced in other cell lines [9]. Subsequently, a validated industrial production process was established. This development was initiated by SIOPEN, a group of international clinical leaders in the field of neuroblastoma and funded by charities throughout Europe. Four European clinical trials with different treatment schedules of ch14.18/CHO are being conducted to investigate the influence of a combined immunotherapy of ch14.18/CHO, interleukin-2 (IL-2) and 13-cis-retinoid acid on the outcome of patients with high-risk NB in the absence or presence of haploidentical blood stem cell transplantation. The first trial established the safety profile of ch14.18/CHO in children with high risk NB [10]. The European phase III clinical trial (HR-NBL 1.5/ESIOP, Eudra CT: 2006-001489-17) and the trial in the context of haploidentical stem cell transplantation (Eudra CT: 2009-015936-14) are based on a short term infusion of 20 mg/m^2^/d ch14.18 over 8 h on five subsequent days. To reduce side effects including neuropathic pain, a Phase I/II clinical trial was initiated based on the same cumulative dose of ch14.18/CHO (100 mg/m^2^/cycle) infused over a longer time period (ten days) (Eudra CT: 2009-018077-3). Within these trial protocols, a set of immune monitoring assays including the detection of ch14.18/CHO serum levels [11] and human anti-ch14.18/CHO immune responses [12], are implemented with the aim to identify immune biomarkers correlating with clinical response to ch14.18/CHO therapy. For a comprehensive assessment, validated bioassays to determine effector functions of ch14.18/CHO namely patient specific ADCC and CDC are of critical importance.

For analysis of patient-specific CDC and ADCC, we established and validated two non-radioactive and non-toxic cytotoxicity assays based on release of acetomethoxy derivate of calcein (calcein-AM), which is a membrane-permeable live-cell labeling dye. With these assays, we demonstrate GD_2_ specific CDC and ADCC activity in ch14.18/CHO treated patients and demonstrated feasibility in the context of multicenter clinical trials.

## Materials and Methods

### Ethic statement

Participants were informed about the testing procedure and gave written informed consent. The study complies with the Declaration of Helsinki and was approved by the ethics committee of the University Medicine of Greifswald, Germany. *De novo* cell line establishment (HGW-1, HGW-2, HGW-3 and HGW-5 cell line) complies with the written informed consent from the donor, parents and legal guardians.

### Tissue culture

Human GD_2_-positive NB cell line LA-N-1 [13] and human melanoma cell line M21 (kindly provided by Prof. R. A. Reisfeld) [14] were cultured in RPMI supplemented with 4.5 g/l glucose, 2 mM stable glutamine (PAA, Pasching, Austria), 10% FCS and 100 U/ml penicillin and 0.1 mg/ml streptomycin (1x P/S; PAA, Pasching, Austria). Human GD_2_-positive NB cell lines CHLA-20 [15], HGW-1, HGW-2, HGW-3 and HGW-5 were cultured in IMDM supplemented with 4 mM stable glutamine, 20% FCS, 1x ITS (BD Biosciences, Heidelberg, Germany) and 1x P/S. Human GD_2_-negative NB cell line SK-N-SH [16] was cultured in DMEM supplemented with 4.5 g/l glucose, 2 mM stable glutamine, 10% FCS and 1x P/S. Hybridoma cells producing ganglidiomab were cultured in DMEM supplemented with 4.5 g/l glucose, 2 mM stable glutamine, 10% FCS, 1x P/S, 1x nonessential amino acids (PAA, Pasching, Austria) and 50 µM β-mercaptoethanol (Sigma Aldrich, Steinheim, Germany).

In order to establish new NB cell lines, tumor tissues and bone marrow samples from NB patients were used. Tumor tissue was first minced followed by homogenisation using 70 µM cell strainer (BD Biosciences, Heidelberg, Germany) and bone marrow samples were treated for 10 min with cold erythrocyte lysis buffer (0.15 M ammonium chloride, 10 mM potassium bicarbonate, 0.1 mM ethylenediaminetetraacetic acid, pH 7.4). After two wash steps (1x PBS, 5 min, 300×g, RT) cell pellets were resuspended in 10 ml 1x PBS and layered over 10 ml of Lymphocyte Separation Medium (PAA, Pasching, Austria). After centrifugation (30 min, 300×g, RT without brake) the upper layer was discarded, a layer of primary cells was carefully transferred into a new tube and the remaining Lymphocyte Separation Medium was washed off with 1x PBS supplemented with 0.5% BSA (Sigma Aldrich, Steinheim, Germany) and 2 mM EDTA (Sigma Aldrich, Steinheim, Germany) (pH 7.4, 10 ml, 5 min, 300×g, RT, brake on). Thereafter, the supernatant was carefully removed and the cell pellet was resuspended with 10 ml 1x PBS/0.5% BSA/2 mM EDTA buffer (pH 7.4) followed by cell counting and evaluation of cell viability. Finally, 1×10^7^ vital cells were used for isolation of GD_2_-positive cell subset with MACS technique (Miltenyi Biotec, Teterow, Germany).

For this purpose 1×10^7^ primary cells were resuspended in 200 µl 1x PBS/0.5% BSA/2 mM EDTA buffer (pH 7.4) and then incubated on ice with 20 µl FcR-blocking reagent (Miltenyi Biotec, Teterow, Germany) for 5 min. Thereafter, the murine anti-GD_2_ Ab 14G2a (1.0 µg) was added followed by incubation for 10 min on ice. After washing with 1x PBS/0.5% BSA/2 mM EDTA buffer (15 ml, pH 7.4, 5 min, 300×g, +4°C) cell pellet was resuspended in 100 µl 1x PBS/0.5% BSA/2 mM EDTA buffer and 20 µl magnetic microbeads conjugated with anti-mouse IgG (Miltenyi Biotec, Teterow, Germany) were added followed by incubation on ice for 15 min. Next, the cells were washed again by adding 15 ml 1x PBS/0.5% BSA/2 mM EDTA buffer (pH 7.4) and centrifuged at 300×g for 5 min at +4°C. 1 ml of 1x PBS/0.5% BSA/2 mM EDTA buffer was used to resuspend the cell pellet and magnetic separation of GD_2_-positive cells was proceeded in MACS-separator (Miltenyi Biotec, Teterow, Germany) by applying the cell suspension onto the LS-separation column (Miltenyi Biotec, Teterow, Germany). The column was washed three times (3 ml, 1x PBS/0.5% BSA/2 mM EDTA buffer, pH 7.4) and then removed from the MACS-separator and placed into a collection tube. Cell fraction containing magnetically labeled GD_2_-positive cells were immediately flushed out by firmly applying the plunger supplied with the column (Miltenyi Biotec, Teterow, Germany). Finally, primary cells were washed with 1x PBS (pH 7.4, 5 min, 300×g, RT) and the cell pellet was resuspended in culture medium as described above.

### Genetic characterization of *de* *novo* neuroblastoma cell lines

To determine DNA profiles of newly established cell lines, short tandem repeats (STR) analysis was done (Eurofins MWG Operon, Ebersberg, Germany). Evaluation of genetic aberrations of each newly established cell line was performed by high resolution SNP array analysis using the Affymetrix ultra HD SNP array (CytoScan HD Array). The combination of copy number with the allele information, i.e. zygosity status, enables the detection of loss of heterozygosity with copy losses as well as copy neutral allelic changes.

### Analysis of neuroblastoma surface markers by flow cytometry

For analysis of GD_2_ and CD56 surface expression, 1×10^6^ cells were stained with 1.0 µg ch14.18/CHO and 0.05 µg anti-CD56-APC (clone HCD56, BioLegend, Fell, Germany) in a total volume of 100 µl for 20 min, on ice, in the dark. The chimeric anti-CD20 antibody rituximab (Roche, Mannheim, Germany) and APC-labeled mouse IgG1 (clone MOPC-21, BioLegend, Fell, Germany) were used as negative controls for ch14.18 and anti-CD56, respectively. Cells were washed once with 1 ml wash buffer (1x PBS, 1% BSA, 0.1% NaN_3_, 0.1% EDTA, pH 7.4) (300×g, 5 min, RT). Supernatant was discarded and cells were incubated with 0.017 µg of PE-labeled anti-human IgG antibody (clone HP6017, BioLegend, Fell, Germany) in a total volume of 100 µl for 20 min, on ice, in the dark. Cells were washed once and resuspended in 500 µl wash buffer for flow cytometric analysis. To exclude dead cells from analysis, 4 µl of a 0.1 mg/ml DAPI solution (Sigma-Aldrich, Steinheim, Germany) were added 5 min prior to acquisition. For each sample 20,000 live cells were analyzed. Sample acquisition was performed at a BD FACS CANTOII using FACSDiva software (BD Biosciences, Heidelberg, Germany) and data was analyzed using FlowJo 7.6.1 software (Treestar, Ashland, OR, USA). For comparison of GD_2_ expression on *de novo* NB cell lines, the ratio of mean fluorescence intensity (MFI) relative to isotype control was calculated according to the formula: geometric fluorescence mean of GD_2_ stained sample/geometric fluorescence mean of isotype control.

### Tyrosine hydroxylase and MDR1 gene expression analysis by RT-PCR

Total RNA was isolated from ≤1×10^7^ cells using the RNA isolation Kit (QIAGEN GmbH, Hilden, Germany) according to the manufacturer's instructions. RNA concentration was determined spectrophotometrically (BioPhotometer plus, Eppendorf, Hamburg, Germany) and 1.0 µg of total RNA was used for cDNA synthesis using qScript cDNA Synthesis Kit (Quanta BioSciences, Gaithersburg, MD, USA) according to the manufacturer’s protocol.

Analysis of human TH (hTH) mRNA expression was performed using gene-specific primers designed with online primer design tool Primer3 and used for PCR. For the detection of hTH mRNA, the forward primer sequence used was 5′-GGC CCA AGG TCC CCT GGT TC-3′ and the reverse primer sequence was 5′-ACA GCA GGC CGG CCA CAG GC-3′. HTH was amplified by 25 cycles consisting of 95°C (30 s) for denaturation, 62°C (30 s) for primer-specific annealing, and 72°C (30 s) for elongation. The PCR product size was 405 bp. PCR without cDNA template (no template control) served as a negative control and GAPDH as a housekeeping gene (forward primer: 5′-GAG TCA ACG GAT TTG GTC GT-3′ and reverse primer: 5′-TTG ATT TTG GAG GGA TCT CG-3′, product size: 238 bp).

To evaluate expression of MDR1 gene, specific primers were designed as described above. Following primer sequences were used for PCR: 5′-GCT CCT GAC TAT GCC AAA GC-3′ (forward primer) and 5′-TCT TCA CCT CCA GGC TCA GT-3′ (reverse primer). MDR1 PCR product (202 bp) was amplified by 25 cycles consisting of 95°C (15 s) for denaturation, 61°C (15 s) for primer-specific annealing, and 72°C (15 s) for elongation. Negative control and GAPDH as an internal control (forward primer: 5′-GAG TCA ACG GAT TTG GTC GT-3′ and reverse primer: 5′-TGT GGT CAT GAG TCC TTC CA-3′, product size: 512 bp) were used as described above. After separation by electrophoresis on 2% agarose gel, PCR products were visualized by ethidium bromide (ImageJ Program). Expression of MDR1 gene product was analyzed relative to the internal control GAPDH.

### Isolation of ganglidiomab from hybridoma supernatants

Ganglidiomab anti-idiotype (anti-Id) Ab was isolated from hybridoma cells as previously described [17]. Briefly, the concentration of ganglidiomab in hybridoma supernatants was determined by ELISA using ch14.18/CHO as a capture mAb [17]. Hybridoma supernatants containing 50 µg/ml of ganglidiomab were filtered and ganglidiomab was concentrated followed by binding to protein G as previously described. After wash steps ganglidiomab was eluted and quantified with ELISA using ch14.18/CHO as a capture mAb [17]. The final product contained 500 µg/ml of ganglidiomab and was used for further assay developments.

### Serum preparation

Blood was collected using BD Vacutainer plastic serum tubes (BD Biosciences, Heidelberg, Germany). Serum was prepared from clotted blood samples by centrifugation for 10 min at 1,700×g, RT and stored in aliquots at −80°C. Serum used for ADCC assays was heat inactivated at 56° for 30 min. Serum as a source for complements in CDC assays was used without further manipulation.

### Isolation of effector cells

#### Isolation of peripheral blood mononuclear cells (PBMCs)

Blood was collected using BD Vacutainer plastic sodium-heparin tubes (BD Biosciences, Heidelberg, Germany) 6 ml of blood were carefully layered over 6 ml of Lymphocyte Separation Medium (PAA, Pasching, Austria). After centrifugation (30 min, 300×g, RT without brake) following four layers were defined: a) a clear upper layer (diluted plasma and platelets), b) a fluffy white layer of PBMCs, c) a clear Lymphocyte Separation Medium layer and d) a lowest substantial pellet of red blood cells containing granulocytes. After the upper layer was discarded, a layer of PBMCs was carefully transferred into a new tube and the remaining Lymphocyte Separation Medium was washed off twice with 1x PBS (10 ml, 5 min, 300×g, RT, brake on). Thereafter, the supernatant was carefully removed and the cell pellet was resuspended with 10 ml 1x PBS followed by counting of PBMCs. To determine relative amounts of lymphocytes, granulocytes and monocytes in PBMCs, cells (2×10^5^) were resuspended in 2 ml 1x PBS and attached to glass slides using cytospin centrifugation (10 min, 200×g, RT). Cytospins were stained using the panoptic method of Pappenheim following the manufacturer’s guidelines and a differential leucocyte count was performed by light microscopy. The lowest layer containing erythrocytes and granulocytes was used for further isolation of granulocytes.

#### Isolation of granulocytes

The lowest layer obtained during the isolation of PMBCs as described above was resuspended in 4 ml of cold erythrocyte lysis buffer (0,15 M ammonium chloride, 10 mM potassium bicarbonate, 0.1 mM ethylenediaminetetraacetic acid, pH 7.4) and incubated for 10 min on ice. Further, cell suspension was centrifuged for 5 min at 300×g (+4°C). Thereafter, the supernatant was carefully removed and the cell pellet was resuspended in 15 ml 1x PBS and centrifuged (5 min, 300×g, +4°C). Then, granulocytes were resuspended in 10 ml 1x PBS and counted. The relative amount of granulocytes was determined by light microscopy following cytospin as described above.

#### Isolation of leukocytes

Leukocytes were isolated from 6 ml of sodium-heparin blood not older than 24 hours using cold erythrocyte lysis buffer. Briefly, 6 ml of whole blood was gently mixed with 12 ml of cold erythrocyte lysis buffer, incubated for 10 min on ice and centrifuged for 5 min at 300×g (+4°C). Thereafter, cell pellet was resuspended in 15 ml 1x PBS and centrifuged again (5 min, 300×g, +4°C). Finally, leukocytes were resuspended in 10 ml 1x PBS and counted.

#### Calcein-AM cytotoxicity assay

In order to evaluate patient-specific ch14.18/CHO-mediated CDC as well as ADCC, two calcein-AM-based release assays were established. For ADCC, NB target cells labeled with membrane-permeable fluorescent dye calcein-AM were incubated with effector cells and serum of treated patients. For CDC, patient serum was incubated with calcein-AM-labeled target cells without effector cells.

Since high spontaneous release of calcein from labeled target cells is an obstacle for calculation of CDC, the level of spontaneous release was analyzed in a panel of GD_2_-positive NB cell lines (LA-N-1, CHLA-20, HGW-1, HGW-2, HGW-3 and HGW-5) and GD_2_-positive melanoma cell line M21. GD_2_-negative NB cell line SK-N-SH served as a negative control. 0.6×10^6^ cells were harvested, counted and washed twice with 10 ml 1x PBS (5 min, 300×g, RT). Cell pellet was then resuspended in 1 ml of respective culture medium and incubated with 10 µM calcein-AM (Sigma Aldrich, Steinheim, Germany) for 30 min at 37°C shaking at 100x rpm under CO_2_-free atmosphere (dark). After two wash steps in serum-free medium, supernatants were collected for background calculation. For spontaneous release determination, 5×10^3^ of calcein-AM labeled target cells were added to each well of a 96-well plate (PAA, Pasching, Austria) and incubated with 12.5% heat-inactivated serum of a healthy donor (30 min, 56°C) for 4 h at 37°C, in the dark. To achieve final volume of 200 µl/well, serum-free cell culture medium (RPMI) was added. For maximum release, target cells were disrupted using ultrasonic homogenizer (30 s, RT). Then, supernatants (50 µl) of each well were transferred to a black 96-well plate for determination of fluorescence at 495 nm excitation and 515 nm emission wavelengths by Synergy HT multimode microplate reader (BioTek Germany, Bad Friedrichshall, Germany). Experiments were analyzed in triplicates using six replicate wells for spontaneous release (target cells only). Spontaneous release in percent was calculated according to the formula: (spontaneous release - background)/(maximum release - background)×100%.

#### Assay conditions for complement-dependent cytotoxicity

In order to determine an optimal dilution factor, serum of a healthy donor was serially diluted to investigate the impact of different serum concentrations on CDC activity. Nine serum concentrations were analyzed: 100%, 50%, 25%, 12.5%, 6.2%, 3.1%, 1.6%, 0.8% and 0.4%. 0.6×10^6^ LA-N-1 cells were labeled with calcein-AM as previously described followed by incubation for 4 h (+37°C, dark) with two defined concentrations of anti-GD_2_ mAb ch14.18/CHO (1.0 and 0.1 µg/ml) prepared using serum of a healthy donor. Additionally, to prove CDC specificity of the target cell lysis, a humanized mAb eculizumab (trade name Soliris; Alexion Europe SAS, Paris, France) selectively inhibiting the cleavage of complement protein C5 to C5a and C5b by the C5 convertase was used. GD_2_-specific CDC of NB cells LA-N-1 was induced by 1.0 µg/ml ch14.18 prepared in serum of a healthy donor (12.5% end concentration). To inhibit ch14.18-mediated CDC, samples were pre-incubated with 1∶10 serial dilutions of 1.0 mg/ml eculizumab prepared in 1x PBS (final concentration: 1000, 100, 10, 1, 0.1 and 0.01 µg/ml). Samples pre-incubated with excess of GD_2_-mimicking anti-idiotype (anti-Id) Ab ganglidiomab (5.0 µg/ml) were included to show GD_2_-specific target cell lysis. MAb rituximab (1.0 µg/ml) was used as a negative control. CDC was calculated according to the formula: (test release - spontaneous release)/(maximum release - spontaneous release)×100%.

To establish a suitable target cell line for calcein-AM-based cytotoxicity assay, selected GD_2_-positive NB cell lines LA-N-1, CHLA-20, HGW-1, HGW-2, HGW-3 and HGW-5 as well as a GD_2_-positive melanoma cell line M21 were used. GD_2_-negative NB cell line SK-N-SH served as a negative control. CDC was performed using 12.5% diluted serum of a healthy donor and 1.0 µg/ml ch14.18/CHO. Rituximab served as a negative control. Additionally, samples pre-incubated with excess ganglidiomab (5.0 µg/ml) were included to show GD_2_-specific target cell lysis. 0.5×10^3^ calcein-labeled cells/well of each target cell line were used and CDC was evaluated as described above.

To show inter-individual variations in CDC activity, serum samples collected from four healthy donors (D1, D2, D3 and D4) were used. CDC was performed using NB cell line LA-N-1 as a target cell line, 1∶8 diluted donor-specific serum (12.5% final concentration) and 1∶10 serial dilutions of 1.0 µg/ml ch14.18/CHO (1,000, 100, 10, 1.0, 0.1 and 0.01 ng/ml).

To analyze CDC-mediated by different anti-GD_2_ mAb (murine 14G2a, chimeric human/murine ch14.18/CHO and IL-2 conjugated humanized hu14.18-IL-2) were used. Serial 1∶2 dilutions of each Ab (final concentration of 1.0, 0.5, 0.25, 0.12, 0.06 and 0.03 µg/ml) were added to calcein-labeled LA-N-1 target cells and incubated with 12.5% serum of a healthy donor for 4 h at 37°C, in the dark. Two anti-GD_2_ Ab lacking binding sites for complement proteins (ch14.18-delta-CH2 and hu14.18 containing mutated Fc part) as well as anti-CD20 mAb rituximab served as negative controls. Moreover, to show GD_2_-dependent specificity of CDC, additional controls containing respective anti-GD_2_ Ab were pre-incubated with excess of GD_2_-mimicking anti-Id Ab ganglidiomab (5.0 µg/ml). CDC was evaluated as previously described.

#### Antibody-dependent cell-mediated cytotoxicity

A systematic analysis of the source of the effector cells and the effector to target (E:T) cell ratio was performed using 10 µg/ml of anti-GD_2_ Ab ch14.18/CHO and LA-N-1 NB cell line as described above. Three effector cell populations (leukocytes, PBMCs and granulocytes) of a healthy donor (D1) were analyzed at different E:T ratios (80∶1, 40∶1 20∶1, 10∶1, 5∶1, 2.5∶1 and 1.25∶1). Additionally, ADCC-mediated by activated effector cells was analyzed. Therefore, effector cells were cultivated for 64 h in RPMI culture medium supplemented with IL-2 (1000 IE/ml; Novartis, Nürnberg, Germany), 4.5 g/l glucose, 2 mM stable glutamine, 10% FCS and 1x P/S. After pre-incubation of labeled target cells (5×10^3^/well) with ch14.18/CHO for 30 min at +37°C (dark), effector cells were added for further 4 h (+37°C, dark). Rituximab served as a negative control. GD_2_-specific ADCC of NB cells was demonstrated by pre-incubation of ch14.18/CHO with excess of anti-Id Ab ganglidiomab (50 µg/ml). To achieve a final volume of 200 µl/well, LA-N-1 cell culture medium was used. Finally, fluorescence levels of supernatants (50 µl) were measured as described above. Experiments were analyzed in triplicates using six replicate wells for spontaneous (target cells only) and three replicates for maximum release. ADCC was calculated according to the formula: (test release - spontaneous release)/(maximum release - spontaneous release)×100%.

To investigate the sensitivity, a concentration range of ch14.18/CHO mAb inducing measurable ADCC, serial Ab dilutions were prepared (10,000, 1,000, 100, 10, 1, 0.1 and 0.01 ng/ml). ADCC evaluation was performed using LA-N-1 as a target cell line and activated leukocytes of a healthy donor (D1) as effector cells at E:T ratio of 40∶1 as described above. Rituximab and ganglidiomab containing controls were included as described above.

#### Assay validation and run acceptance analysis

To show reproducibility, accuracy and precision of the established cytotoxicity assay, both within-assay and inter-assay precision analysis were determined according to international guidelines for bioanalytical method validation [18]. For both data validation and run acceptance analysis, we included in each analytical run a quality control containing a known concentration of ch14.18/CHO mAb (1.0 µg/ml) prepared in serum of a healthy donor (D1).

Within-assay precision was determined with ten triplicated samples containing 1.0 µg/ml ch14.18/CHO in serum of a healthy donor. The within-assay precision value was calculated as follows: (standard deviation (SD) of replicates divided by mean of replicates)×100%. To confirm consistency of measurements over time, the inter-assay precision was calculated. For this, ten triplicated samples containing a defined concentration of ch14.18/CHO (1.0 µg/ml) prepared in serum of a healthy donor were analyzed by different operators on different days. The inter-assay precision was calculated according to the formula: (SD of replicates divided by mean of replicates)×100%.

For validation of cytotoxicity analysis, we developed a set of tailored QCs containing defined concentrations of ch14.18/CHO (1.0 µg/ml) prepared in serum of a healthy donor. For each serum batch the level of cytotoxicity was first determined in at least six independent measurements by different operators on different days. Then, we calculated the CV for these assays and plotted the results over time. Only the serum batch spiked with ch14.18/CHO showing CV under 20% was used as QC and included in each analytical run for run acceptance analysis.

To accept or reject analytical runs, QCs were included in each run. Deviation of the QC results from their nominal value was calculated as absolute value (modulus) using the formula: | 100% - (MEAN/nominal value)×100% |, according to international guidelines for bioanalytical method validation [18]. If the differences between the QC and its respective nominal values exceeded 20%, the run was rejected and the data were not included in the data analysis. Only runs including QC deviating from the nominal within 20% were accepted.

#### Evaluation of CDC and ADCC in neuroblastoma patients

The established cytotoxicity assay was applied to serum and sodium-heparin blood samples collected from three selected NB patients (patient 1, patient 2, patient 3) treated in a European anti-GD_2_ immunotherapy study with ch14.18/CHO in combination with IL-2. Patients received 6×10^6^ IU/m^2 ^s.c. IL-2 (day 1–5; 8–12) and ten day long-term infusion (LTI) of 100 mg/m^2^ ch14.18/CHO (day 8–17). CDC and ADCC were determined at two time points: prior to start of treatment (day 1) and on day 15 as described above.

#### Stability of CDC and effector cell viability

To evaluate the impact of shipping condition on CDC, viability and cell count of effector cells, we analyzed both serum and whole blood samples of a healthy donor (D1) stored under different conditions or subjected to five freeze-thaw cycles. The serum samples were prepared as described above. For evaluation of repeated freezing and thawing or storing at RT (up to 96 h), serum samples aliquots containing two defined ch14.18/CHO concentrations (1 and 0.1 µg/ml) were used. For evaluation of leukocyte viability and cell count, whole blood samples were collected in either BD Vacutainer plastic K2EDTA- (BD Biosciences, Heidelberg, Germany) or BD Vacutainer plastic sodium-heparin-containing tubes (BD Biosciences, Heidelberg, Germany) and stored at RT for up to 72 h. CDC was evaluated using the established cytotoxicity assay. Rituximab containing samples and Ab-free sera were used as negative controls. Viability of leukocytes was evaluated using trypan blue test and a cell count with automated cell counter (EMD Millipore Corporation, Billerica, MA, USA).

#### Statistics

After testing for normality and equal variance across groups, differences between groups were assessed with the Students *t*-test. A P level of < 0.05 was considered significant. All data are given as means ± SD or means ± SEM. Analysis was performed using the software SigmaStat (Jandel, San Rafael, CA).

## Results

### Target cell line selection to establish CDC and ADCC bioassays

The selection of a suitable target cell line to set up immune bioassays for the monitoring of clinical trials was done with a panel of seven NB cell lines. The panel consisted of three cell lines propagated for many years (LA-N-1, CHLA-20, and SK-N-SH) and of four lines newly established from patients with relapsed NB. This was accomplished by isolation of GD_2_-positive cells from primary tumor tissue or bone marrow by MACS and culture in IMDM-based medium as described in Materials and Methods. Newly established cell lines were named HGW-1 (bone marrow originated), HGW-2 (primary tumor originated), HGW-3 (primary tumor originated) and HGW-5 (bone marrow originated). HGW-1,2 and 5 cell lines were MYCN amplified and positive for 17 q gain (Table 1). HGW-3 and HGW-5 cells also showed the typical deletion of chromosome 1p. HGW-1 showed gain of 11q in contrast to the other lines (Table 1). Analysis of HGW-2 is pending. Finally, cell line DNA profiles determined with STR analysis clearly showed the *de novo* nature of each cell line.

**Table 1:** Genetic aberrations of *de novo* neuroblastoma cell lines.

	MYCN amplification	NMYC copy number	1p deletion	11q gain	17q gain
HGW-1	+	36	−	+	+
HGW-2	N.D.	N.D.	N.D.	N.D.	N.D.
HGW-3	+	14	+	−	+
HGW-5	+	25	+	−	+

Genetic aberrations of *de novo* neuroblastoma cell lines were evaluated using high resolution SNP array analysis. N.D; not done.

Further characterization of these new HGW cell lines were done in comparison to NB cell lines LA-N-1, CHLA-20 and SK-N-SH as well as a melanoma cell line M21.

Since GD_2_ is the target antigen of ch14.18, its expression on the target cell line is crucial to set up CDC and ADCC bioassays. The GD_2_ expression analysis was complemented with CD56 since both surface molecules are typical features of NB cells.

**Figure 1:**
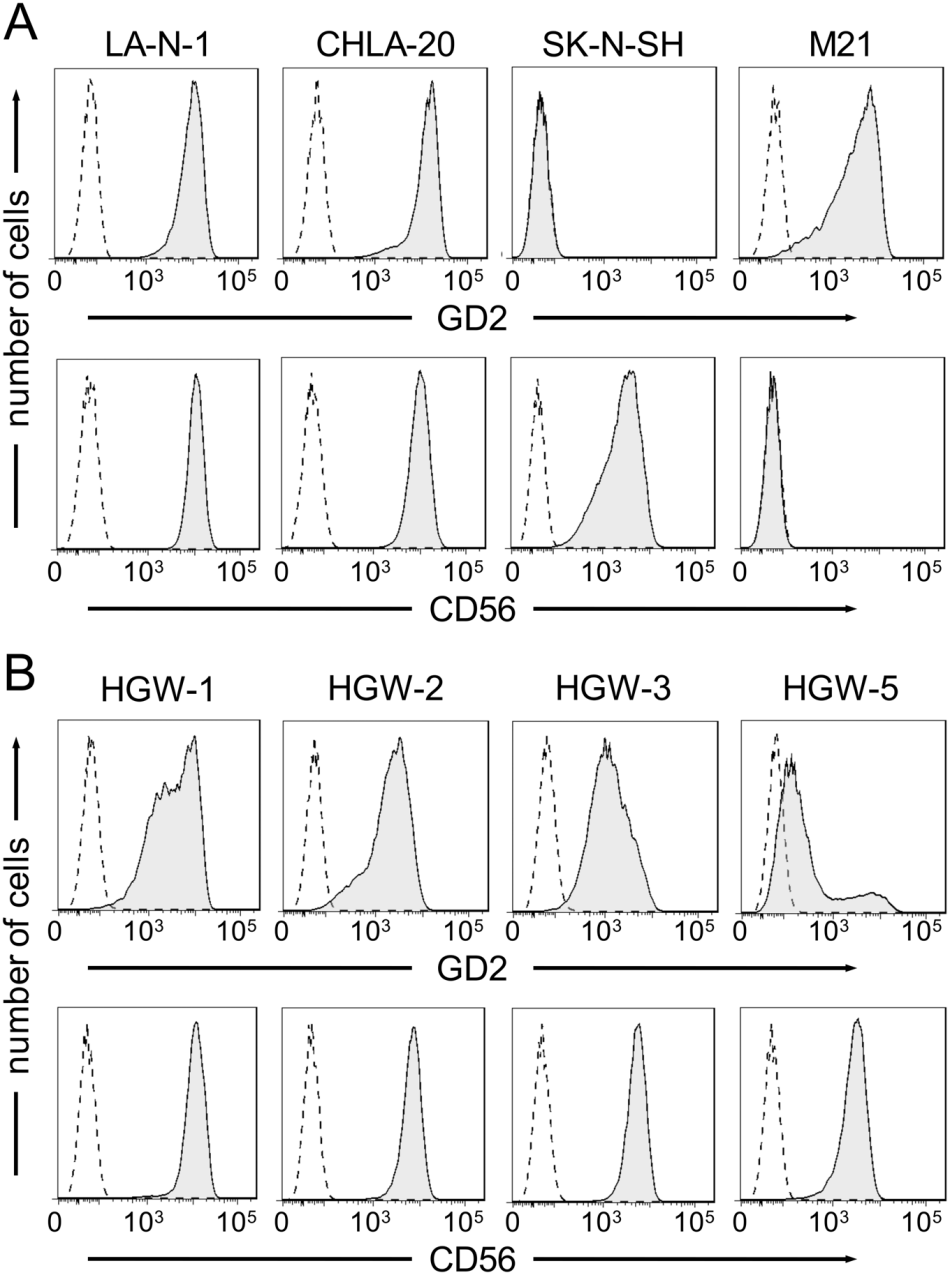
Flow cytometric analysis of GD_2_ and CD56 expression. NB cells were stained with 1.0 µg ch14.18/CHO and 0.05 µg anti-CD56-APC, followed by incubation with 0.017 µg of PE-labeled anti-human IgG secondary antibody. Chimeric anti-CD20 antibody and APC-labeled mouse IgG1 were utilized as isotype controls, respectively. (**A**) Expression of GD_2_ (upper panel, black filled curve) and CD56 (lower panel, black filled curve) on NB cell lines LA-N-1, CHLA-20, SK-N-SH and the melanoma cell line M21. Respective isotype controls are indicated in both panels as black dashed curves. Results are presented as representative histograms. (**B**) Selected histograms of GD_2_ (upper panel, black filled curve) and CD56 (lower panel, black filled curve) expression on *de novo* NB cell lines HGW-1, HGW-2, HGW-3 and HGW-5. Respective isotype controls are shown as black dashed curves in both panels.

NB origin of newly established cell lines HGW-1, HGW-2, HGW-3 and HGW-5 was confirmed by detection ob both CD56 and GD_2_ NB expression markers (Fig. 1B). All *de novo* cell lines showed homogeneous expression of CD56 and heterogeneous expression of GD_2_ (Fig. 1B). To compare GD_2_ expression profiles, MFI ratios were used. The highest MFI ratio was found on HGW-1 (63.4). HGW-2 and -3 displayed MFI ratios of 46.9 and 22.9, respectively. The lowest MFI ratio was calculated for HGW-5 (5.0). Interestingly, GD_2_ expression analysis of HGW-5 revealed two distinct subpopulations. About 84% of cells had a GD_2_^dim^ phenotype (MFI ratio 3.0) and the smaller subpopulation of about 16% had a GD_2_^bright^ expression pattern (MFI ratio 80.5; Fig. 1B). In contrast, HGW-3 cell line which consists of two morphologically distinctive cell populations (adherent and suspension cells) did not show differences in GD_2_ expression profiles.

The NB cell lines LA-N-1 and CHLA-20 used as controls revealed high expression of both NB marker GD_2_ and CD56 (Fig. 1A). In contrast, SK-N-SH showed high expression of CD56 but lacked expression of GD_2_ (Fig. 1A), therefore, SK-N-SH was excluded from consideration as a target cell line for the bioassays. As expected, a melanoma cell line M21 did not show CD56 expression but a high expression of GD_2_ (Fig. 1A).

**Figure 2:**
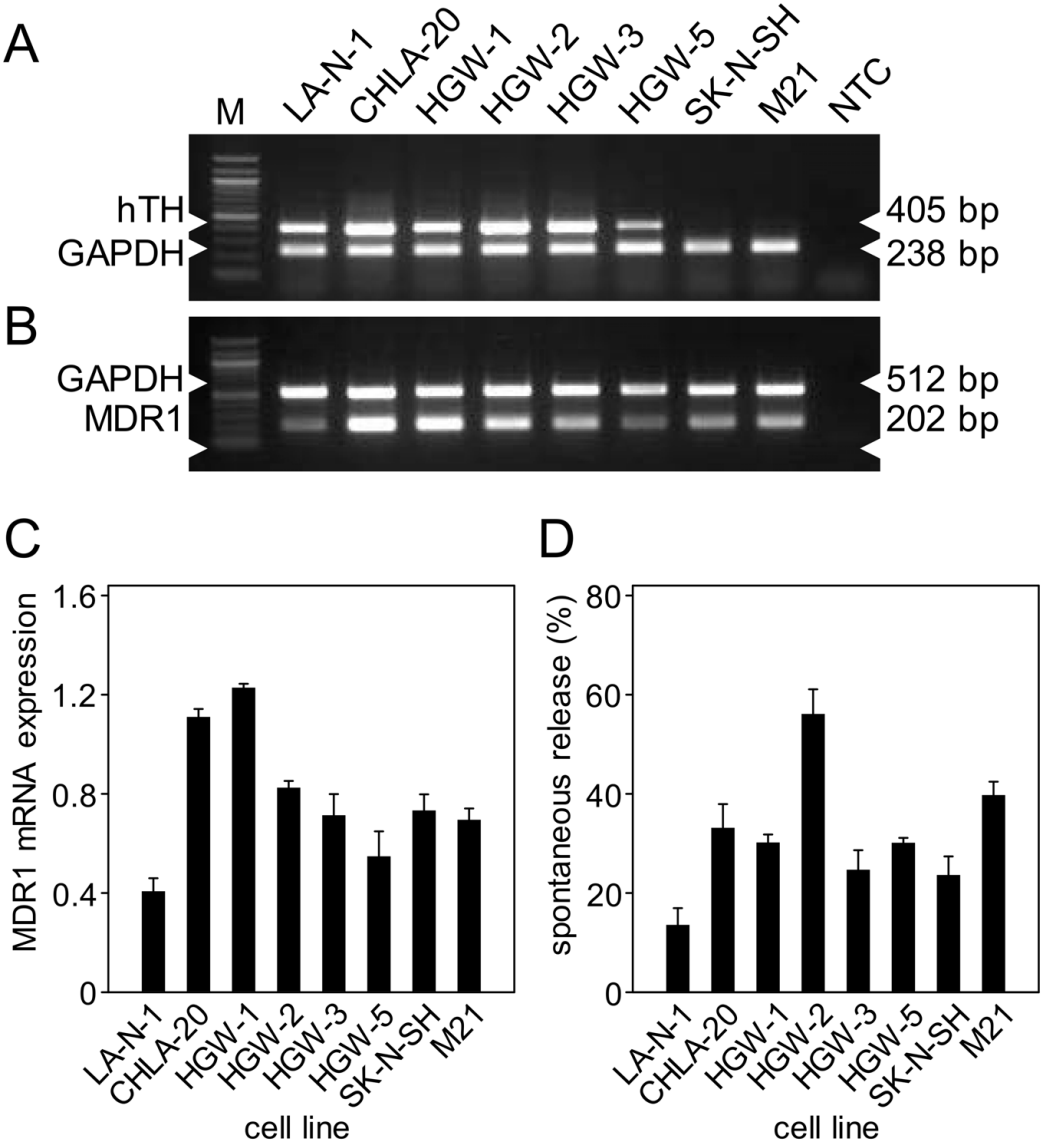
RT-PCR analysis of human tyrosine hydroxylase and MDR1 mRNA expression and level of spontaneous release of calcein from NB cell lines. RNA of human NB cell lines LA-N-1, CHLA-20, HGW-1, HGW-2, HGW-3 and HGW-5 was used for RT-PCR analysis of hTH (**A**) and MDR1 (**B**) gene expression (PCR-product sizes 405 bp and 202 bp, respectively). Expression of GAPDH mRNA served as internal control. (**C**) Densitometric analysis of MDR1 mRNA expression relative to GAPDH. Values are given as means ± SE of three independent experiments. HTH-negative NB cell line SK-N-SH and melanoma cell line M21 served as negative controls for hTH mRNA RT-PCR analysis. NTC, no template control. (**D**) Levels of spontaneous release of calcein from NB cells examined after 4 h incubation of calcein-labeled cells with 12.5% heat inactivated serum.

In order to further characterize the newly established HGW cell lines, we also investigated expression of hTH, the first step enzyme of catecholamine biosynthesis, which is a typical feature of NB cells. Expression of hTH mRNA was found in all newly established NB cell lines HGW-1, HGW-2, HGW-3 and HGW-5 as well as in NB cell lines LA-N-1 and CHLA-20 (Fig. 2A) in contrast to the melanoma cell line M21 used as a negative control. The NB cell line SK-N-SH was surprisingly hTH negative further supporting the decision to exclude this cell line from further consideration.

The analysis of MDR1 gene expression was used as further selection criterion since calcein is an MDR1 substrate. Therefore, high MDR1 expression results in active transport of calcein into the extracellular space which accounts for a high background in calcein release assays [19]. The weakest mRNA expression of MDR1 was found in LA-N-1. (Fig. 2B–C). CHLA-20 and HGW-1 showed about three-fold and SK-N-SH, M21, HGW-2 and -3 about two-fold higher levels of MDR1 mRNA expression compared to LA-N-1 (Fig. 2B–C). These findings correlate with spontaneous release data for cell lines analyzed revealing the lowest level of spontaneous release in LA-N-1 cells (Fig. 2D).

### Assessment of variables for the complement-dependent cytotoxicity bioassay

**Figure 3:**
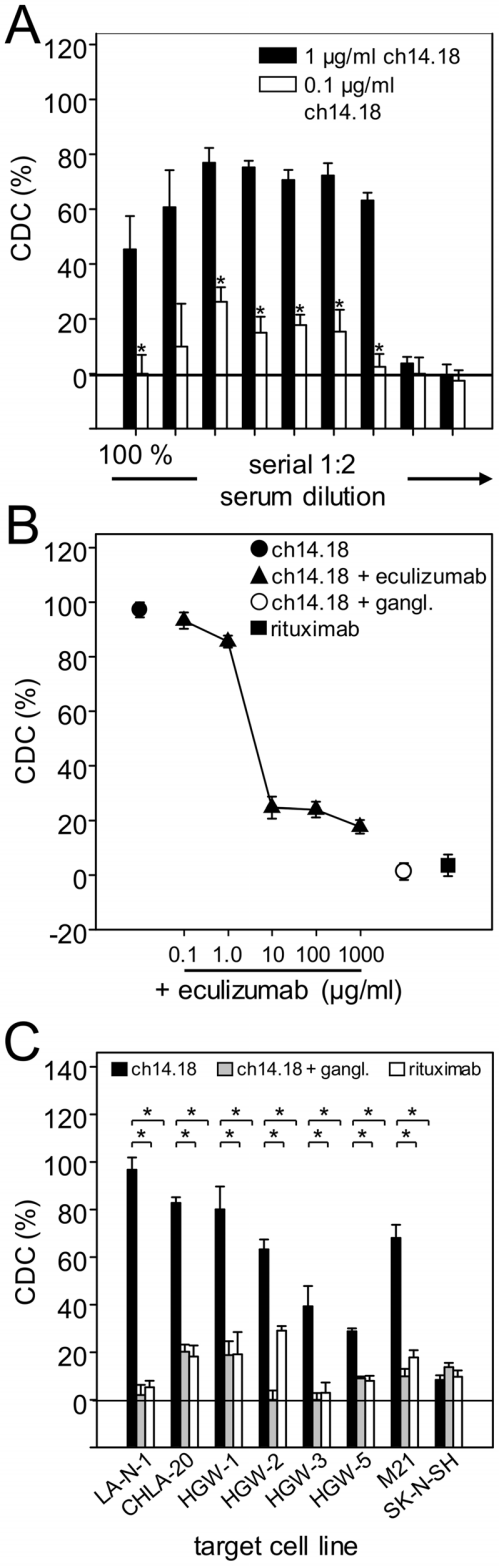
Assessment of variables for the complement-dependent cytotoxicity bioassay. (**A**) Analysis of CDC mediated by different serum concentrations (100%, 50%, 25%, 12.5%, 6.3%, 3.1%, 1.6%, 0.8%, 0.4%) using NB cell line LA-N-1 as a target cell line and two defined concentrations of anti-GD_2_ Ab ch14.18/CHO (1.0 µg/ml (black column) and 0.1 µg/ml (white column). (**B**) Concentration-dependent inhibition of CDC mediated by 1.0 µg/ml ch14.18/CHO (closed circle) by complement inhibitor eculizumab (closed triangles). Pre-incubation with excess of anti-Id Ab ganglidiomab (5.0 µg/ml; open circle) was performed to show GD_2_-specific target cell lysis. Rituximab (1.0 µg/ml; closed square) served as a negative control. (**C**) Evaluation of CDC mediated by different NB cell lines, 12.5% serum and anti-GD_2_ Ab ch14.18/CHO (1 µg/ml; black column). Ganglidiomab (5.0 µg/ml; grey columns) and rituximab (1.0 µg/ml; white columns) served as controls. Data are shown as mean values ± SE of three independent experiments performed at least in triplicates. *t*-test; *P < 0.05.

The optimal serum dilution used for the CDC bioassay with patient samples was evaluated by using a serial 1∶2.dilution (undiluted, 1∶2, 1∶4, 1∶8, 1∶16, 1∶32, 1∶64, 1∶128, 1∶256) of serum of a healthy donor in the presence and absence of two distinct concentrations of anti-GD_2_ Ab ch14.18/CHO, i.e. 1.0 and 0.1 µg/ml, respectively. Interestingly, we found a bell-shaped curve of ch14.18-mediated CDC activity with decreasing serum concentrations at both ch14.18 concentration levels (Fig. 3A). CDC-mediated by 1.0 µg/ml ch14.18/CHO (Fig. 3A, black columns) revealed a maximum of about 80% target cell lysis in a serum dilution range from 1∶4 to 1∶64. In contrast, addition of undiluted or 1∶2, 1∶128 and 1∶256 diluted serum resulted in lower CDC activity levels (45%, 65%, 4% and 4%, respectively). A similar pattern was observed with 0.1 µg/ml ch14.18/CHO. At this antibody concentration, CDC-mediated a maximum of about 30% target cell lysis at serum dilution of 1∶4, 1∶8, 1∶16 and 1∶32 (Fig. 3A, white columns). Again, the undiluted as well as 1∶128 and 1∶256 diluted sera (final concentrations: 3.1% and 1.6%, respectively) could not induce measurable CDC-mediated target cell lysis, and CDC-mediated by 1∶2 diluted serum was found to be only at 14%.

In summary, the highest CDC-mediated target cell lysis was observed by both concentrations of ch14.18/CHO (1.0 and 0.1 µg/ml) to be between 1∶4 and 1∶32. Based on these data we defined the serum dilution at 1∶8 as the standard procedure to evaluate CDC in treated patients.

To further confirm that the cytotoxic activity observed in these experiments is mediated by the complement system, we added the complement inhibitor eculizumab (trade name Soliris) to our system (Fig. 3B, closed triangles). Ch14.18 (1.0 µg/ml) prepared in serum of a healthy donor (D1) (12.5% final concentration) induced a LA-N-1 target cell lysis of 100%. Co-incubation with a serial dilution of 1.0 mg/ml eculizumab inhibited the cytotoxic activity in a concentration-dependent manner resulting in almost complete abrogation of ch14.18-mediated target cell lysis (Fig. 3B, closed triangles). These findings prove the complement-mediated cytotoxic effect. GD_2_ specificity was also demonstrated by pre-incubation of ch14.18 with excess of anti-Id Ab ganglidiomab (Fig. 3B, open circle) or by replacing ch14.18 with similar concentration of mAb rituximab used as a negative control (Fig. 3B, closed square).

In order to make a final decision on the target NB cell line to be used for the bioassays, we analyzed CDC against our panel of cell lines with 12.5% serum (1∶8 dilution) as a source of complement and 1.0 µg/ml ch14.18/CHO (Fig. 3C). The highest level of ch14.18/CHO-mediated CDC was observed using LA-N-1 (97%) NB cells compared to all other cell lines analyzed in particular HGW-5. These differences in GD_2_-specific lysis correlate with GD_2_ expression profiles of NB cell lines analyzed with flow cytometry (Fig. 1B). GD_2_ negative SK-N-SH cells were used as a negative control. Importantly, pre-incubation with excess of anti-Id ganglidiomab resulted in complete abrogation of CDC in that cell line. Interestingly, for some cell lines, we found antibody-independent target cell lysis in samples incubated with 1.0 µg/ml non-specific rituximab or excess of anti-Id ganglidiomab (CHLA-20, HGW-1, HGW-5 and M21), indicating the activation of Ab-independent alternative complement activation pathways. In summary, LA-N-1 showed the highest sensitivity and specificity for GD_2_-specific CDC-mediated by ch14.18/CHO which was therefore selected as the designated cell line to establish the bioassays.

**Figure 4:**
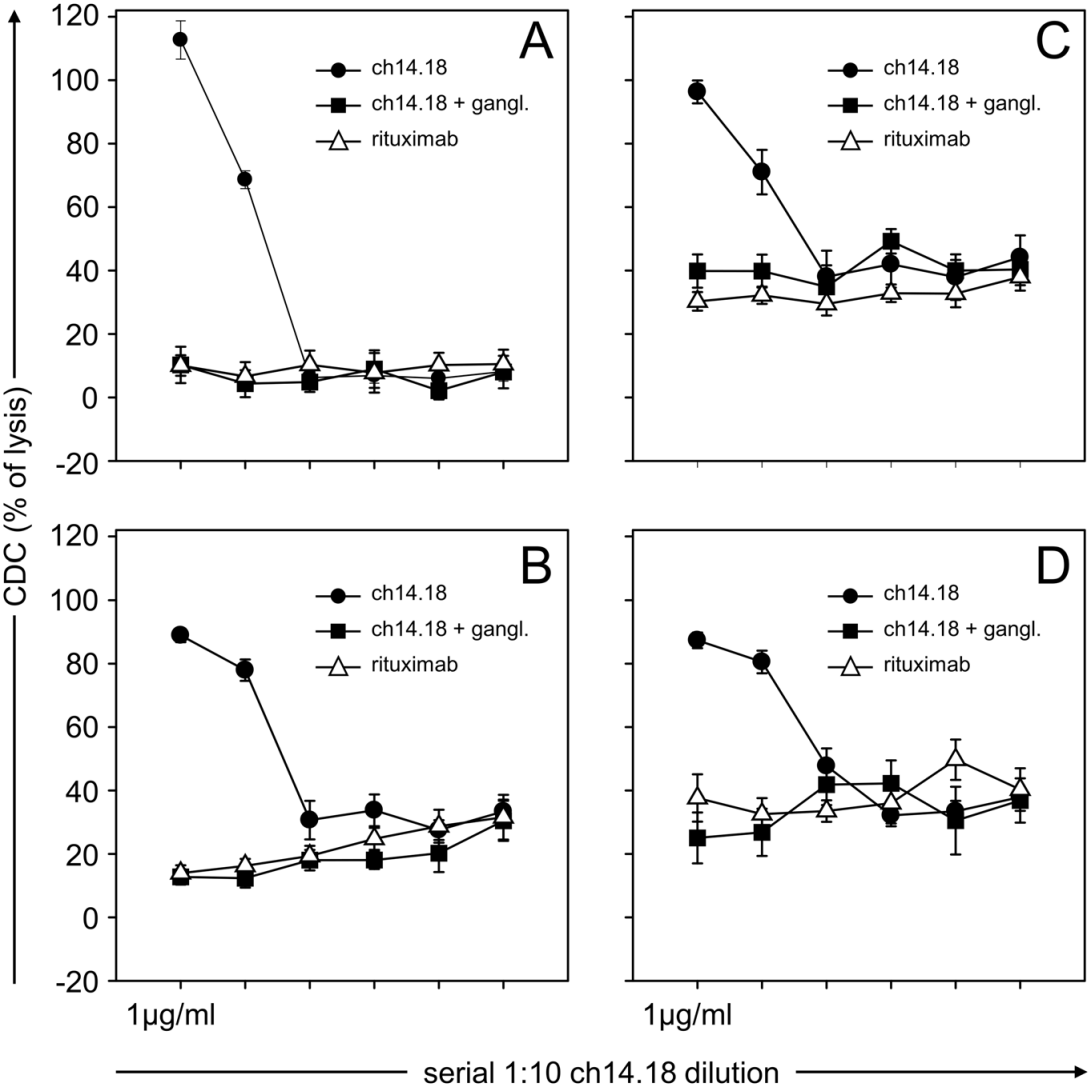
Evaluation of donor-specific CDC. Serum samples (12.5% final concentration) of four healthy donors D1 (**A**), D2 (**B**), D3 (**C**) and D4 (**D**) were analyzed using calcein-AM-based cytotoxicity assay as described in section “Materials and Methods”. Serial dilutions of 1.0 µg/ml ch14.18/CHO were used for CDC (closed circles). Rituximab served as a negative control (1 µg/ml; open triangles). To show GD_2_-specific target cell lysis, samples were pre-incubated with excess of GD_2_-mimicking anti-Id Ab ganglidiomab (5 µg/ml; closed squares). Data are shown as mean values ± SE of three independent experiments performed at least in triplicates.

The impact of the source of complement on ch14.18/CHO-mediated CDC against LA-N-1 NB target cells was analyzed using sera of four healthy donors (D1–4) (Fig. 4) and compared to that of rituximab used as a negative control. The cytotoxic activity mediated by 1.0 µg/ml ch14.18/CHO did not reveal significant inter-individual variations with a maximum activity between 90–100%. GD_2_ specificity was demonstrated by a complete inhibition of CDC-mediated cytotoxicity to the level of the negative control (rituximab) after pre-incubation of serum samples with 5.0 µg/ml anti-Id Ab ganglidiomab. Interestingly, different inter-individual levels of ch14.18/CHO-independent anti-tumor cytotoxicity could be observed in serum samples of the selected donors in a range of 10% to 40%, indicating the presence of natural CDC activity against LA-N-1 as a result of complement activation mediated by the non-classical pathway [20]. Ch14.18/CHO concentrations below 10 ng/ml did not mediate detectable CDC in all donor sera analyzed. Based on these data we used for further experiments serum of a donor D1 lacking Ab-independent cytotoxicity (Fig. 4A).

**Figure 5:**
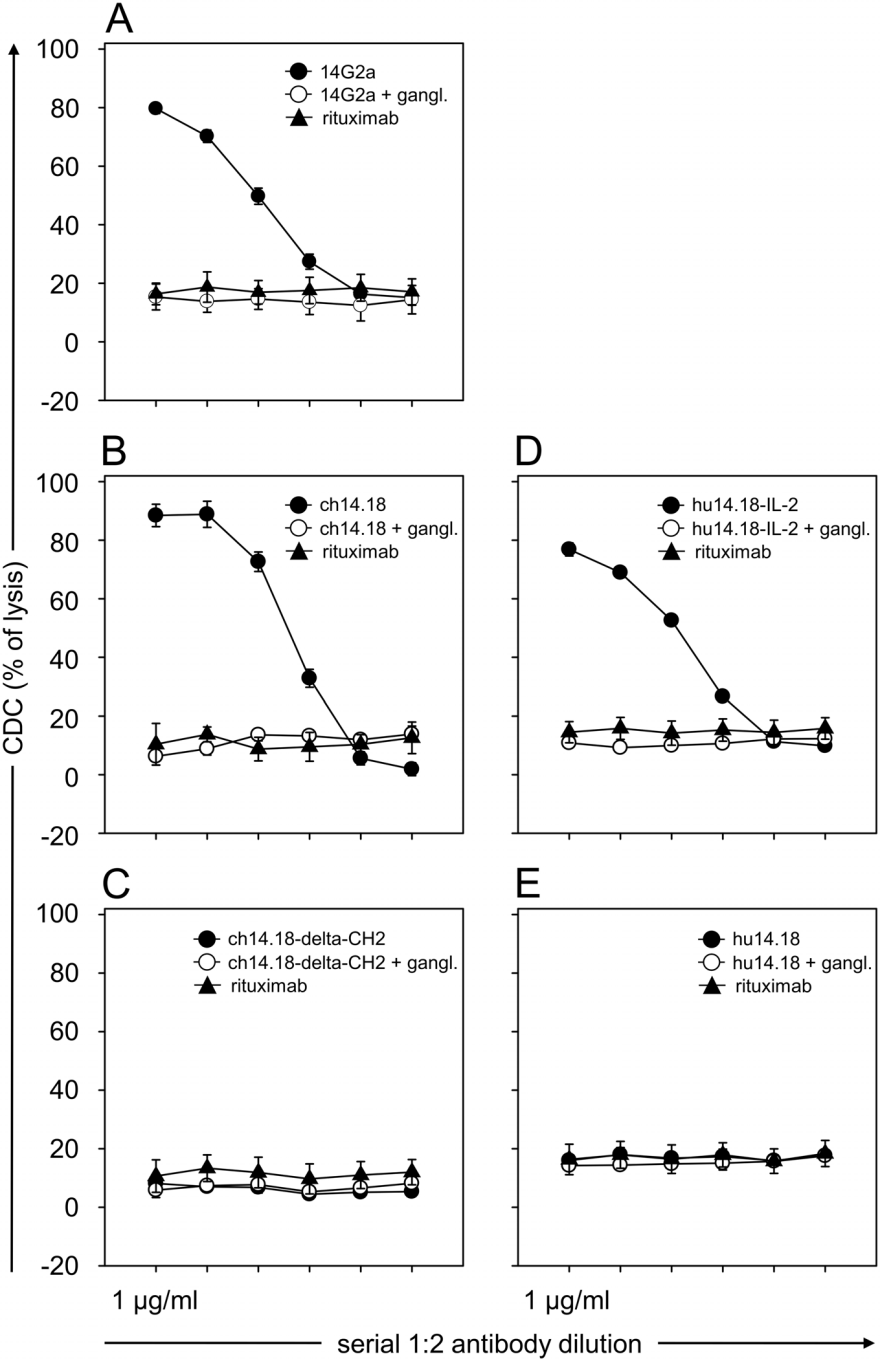
Evaluation of CDC-mediated by different anti-GD_2_ Ab. Serial dilutions (final concentration of 1.0, 0.5, 0.25, 0.12, 0.06 and 0.03 µg/ml) of murine 14G2a (**A**, closed circles), chimeric human/murine ch14.18/CHO (**B**, closed circles), and IL-2 conjugated humanized hu14.18-IL-2 (**D**, closed circles) were used for CDC. Complement fixation deficient mutants chimeric ch14.18-delta-CH2 (**C**, closed circles) and humanized hu14.18 (**E**, closed circles) as well as rituximab (closed triangles) served as negative controls. To show GD_2_-dependent specificity of CDC induced, additional controls containing respective anti-GD_2_ Ab were pre-incubated with excess of GD_2_-mimicking anti-Id Ab ganglidiomab (5 µg/ml; open circles). Data are shown as mean values ± SE of three independent experiments performed at least in triplicates.

CDC-mediated by different anti-GD_2_ mAb constructs including 14G2a, ch14.18/CHO, hu14.18-IL-2 was compared to complement fixation deficient mutants hu14.18 K322 [21] and ch14.18-delta-CH2 against LA-N-1 NB target cells using serum of a healthy donor D1 described above (Fig. 5). The level of a concentration-dependent CDC activity observed was highest with ch14.18/CHO followed by 14G2a and hu14.18-IL2 (Fig. 1B and D) compared to rituximab negative controls. The activity was GD_2_-specific since pre-incubation of samples with 5.0 µg/ml anti-Id ganglidiomab completely inhibited target cell lysis (Fig. 5). As expected, no detectable CDC could be observed with anti-GD_2_ antibody constructs which lack complement binding sites (ch14.18-delta-CH2 and hu14.18 K322) (Fig. 5C and E). In summary, the CDC assay revealed consistent GD_2_-specific results related to the presence and absence of a complement fixation moiety within the anti-GD_2_ construct, with the highest level for ch14.18/CHO.

### Antibody-dependent cell-mediated cytotoxicity bioassay

**Figure 6:**
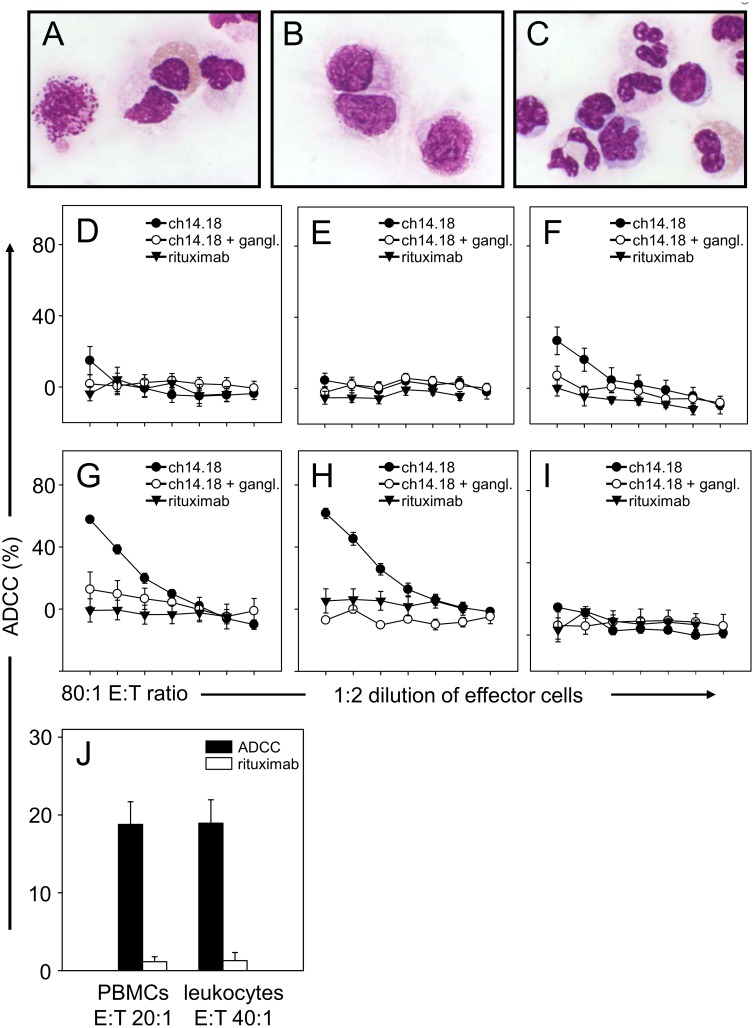
Evaluation of ADCC-mediated by different effector cell subsets. Three populations of freshly isolated effector cells of a healthy donor were compared for ADCC: leukocytes (**A**), PBMCs (**B**) and granulocyte rich fraction (**C**). ADCC were performed using calcein-AM-based cytotoxicity assay as described in Materials and Methods. Effector cells were incubated with 10 µg/ml ch14.18/CHO (closed circles) and calcein-labeled target cells LA-N-1 at different E:T ratios (80∶1, 40∶1, 20∶1, 10∶1, 5∶1, 2.5∶1 and 1.25∶1) and ADCC mediated by leukocytes (**D**), PBMCs (**E**) and effector cells of granulocytes rich fraction (**F**) were examined. In order to simulate the use of IL-2 in combination with ch14.18/CHO in clinical trials, effector cells were incubated for 64 h in cell culture medium supplemented with 1,000 IU/ml IL-2. ADCC mediated by IL-2 treated leukocytes (**G**), IL-2 treated PBMCs (**H**) and IL-2 treated cells of granulocyte rich fraction (**I**) were calculated as described in Materials and Methods. Rituximab served as a negative control (closed triangles). GD_2_-specific ADCC of NB cells was demonstrated by pre-incubation of ch14.18/CHO with excess of anti-Id Ab ganglidiomab (open circles). Data are shown as mean values ± SE of three independent experiments performed at least in triplicates. (**J**) Comparison of ADCC mediated by leukocytes at E:T ratio of 40∶1 and PBMCs at E:T ratio 20∶1 isolated from the same blood sample of five selected NB patients treated with a combination of IL-2 and ch14.18/CHO (black columns). Rituximab served as a negative control (white columns).

The analysis of ADCC was also performed using LA-N-1 NB target cells labeled with calcein as described in Materials and Methods. Assay conditions related to effector cell preparation and determination of an optimal effector-to-target cell ratio were done with blood samples of a healthy donor D1 in the presence of 10 µg/ml ch14.18/CHO compared to rituximab and to samples incubated with ch14.18/CHO and anti-Id used as negative controls (Fig. 6A–I) as well as patients treated with ch14.18/CHO (Fig. 6J), respectively.

For the analysis of ch14.18/CHO-mediated ADCC, three distinct sources of effector cells were systematically evaluated. The first source consisted of a leukocyte preparation following red blood cell lysis of anti-coagulated blood (Fig. 6C). The second and third source was prepared from blood samples centrifuged in a blood separation media as described in Materials and Methods. Lymphocyte rich PBMCs were collected from the fluffy white layer (Fig. 6B) and a granulocyte rich cell fraction was collected from the lowest layer followed by lysis of erythrocytes as described above (Fig. 6A). Additionally, amounts of lymphocytes, granulocytes and monocytes were determined in both PBMC and granulocyte cell fractions showing about 80% lymphocytes, 10% granulocytes and 10% monocytes and 25% lymphocytes, 72% granulocytes and 3% monocytes in PBMC and granulocyte rich fraction, respectively.

The initial evaluation included such effector cells prepared from a healthy donor D1 either used immediately after isolation (0 h; Fig. 6D–F) or after incubation in medium containing 1,000 IU/ml IL-2 for 64 h (Fig. 6G–I). The IL-2 incubation was done in order to simulate the use of IL-2 in combination with ch14.18/CHO in clinical trials.

Of the fresh isolated effector cell preparations used immediately after isolation, only the granulocyte rich cell fraction mediated a significant ADCC reaction at an E:T ratio of 80∶1. This effect was GD_2_-specific since anti-Id ganglidiomab completely inhibited ch14.18-mediated ADCC (Fig. 6F). In contrast, both freshly isolated leukocytes and lymphocyte rich PBMCs did not show significant cytotoxic activity against LA-N-1 NB target cells at any E:T ratio (Fig. 6D–E). This sharply contrasts to the response observed following IL-2 incubation (Fig. 6G–H). A strong increase of ADCC-mediated by leukocytes and lymphocyte rich PBMCs (Fig. 6G–H) but not by the granulocyte rich fraction was observed (Fig. 6I). All responses were GD_2_-specific and correlated with the E:T ratio. In conclusion, leukocytes prepared by red blood cell lysis of anti-coagulated blood and the lymphocyte rich PBMC preparations revealed equivocal ADCC responses at an E:T ratio of 20∶1 and 40∶1.

In order to confirm these results, we isolated leukocytes and lymphocyte rich PBMC preparations from the same blood sample obtained from five NB patients treated with a combination of IL-2 and ch14.18/CHO (Fig. 6J). Again, both effector cell preparations revealed inter-exchangeable results when used at an E:T ratio of 20∶1 (PBMCs) and 40∶1 (leukocytes) in contrast to rituximab controls, respectively, which in conclusion are both appropriate for immune monitoring.

**Figure 7:**
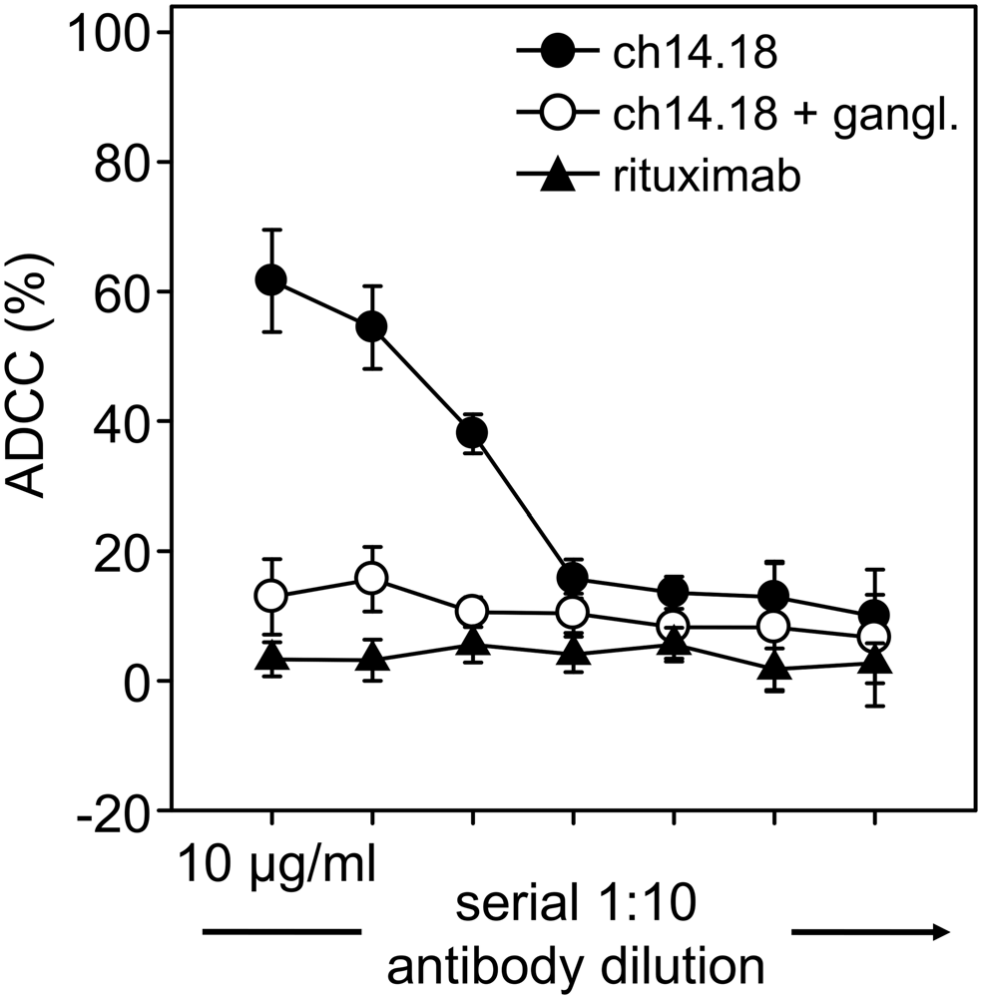
Determination of ADCC sensitivity. Different concentrations of anti-GD_2_ mAb ch14.18/CHO (10,000, 1,000, 100, 10, 1, 0.1 and 0.01 ng/ml, closed circles) were used to investigate a concentration range of Ab inducing a detectable ADCC. Activated leukocytes of a healthy donor were incubated with calcein-labeled LA-N-1 cells at E:T ratio of 40∶1. Rituximab (closed triangles) and anti-Id Ab ganglidiomab (open circles) containing controls were included as described in Materials and Methods. Data are shown as mean values ± SE of three independent experiments performed at least in triplicates.

The sensitivity of the ADCC reaction was determined using leukocytes prepared by red blood cell lysis of anticoagulated blood of a healthy donor (D1) cultivated in medium containing 1,000 IU/ml IL-2 for 64 hours at an E:T ratio of 40∶1 in the presence of varying concentrations of ch14.18/CHO (Fig. 7). A concentration-dependent ADCC was observed with a limit of detection at 10 ng/ml ch14.18/CHO. Again, the response was GD_2_-specific, since pre-incubation with anti-Id ganglidiomab inhibited the cytotoxic activity to the level of background responses observed with rituximab used as a negative control.

### Validation of bioassays, quality controls, run acceptance criteria and sample stability

**Figure 8:**
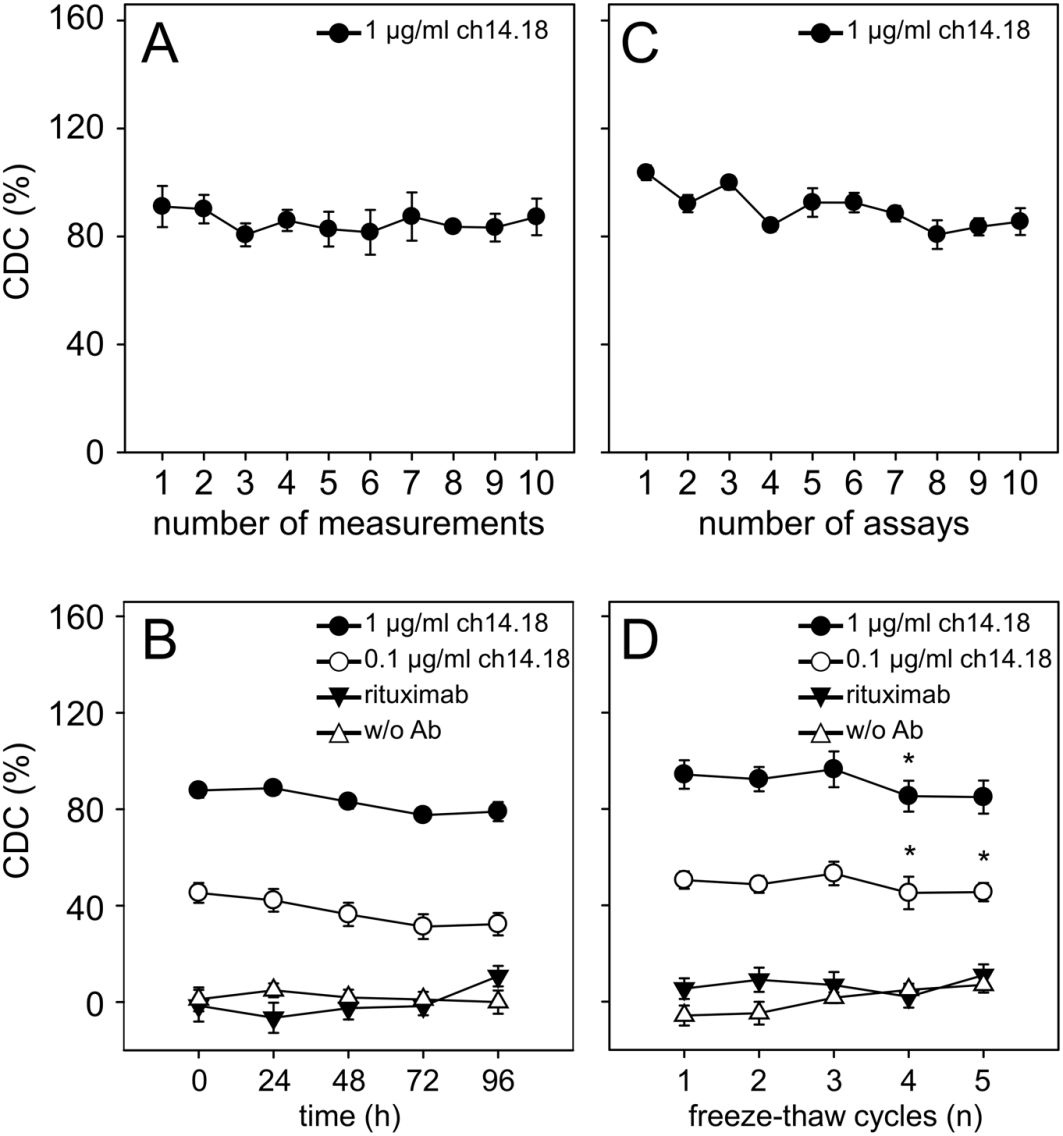
Within-assay precision, inter-assay precision and sample stability. For reliable and reproducible evaluation of cytotoxicity, within-assay and inter-assay precision analyses were performed (**A** and **C**). Both parameters were calculated according to the formula: SD/mean×100% and found to be under 20%. To determine within-assay precision (**A**), triplicated serum samples of a healthy donor (12.5% final concentration) with defined ch14.18/CHO concentrations (1.0 µg/ml) and calcein-labeled LA-N-1 target cells were analyzed. The cytotoxicity analysis was repeated ten times on the same plate. Results are presented as mean CDC of triplicates ± SD for ten data sets. Inter-assay precision (**C**) was determined on different days by different operators. Ten independent measurements of serum samples containing 1.0 µg/ml ch14.18/CHO and calcein-labeled LA-N-1 target cells were performed. The analyzed cytotoxicity levels are given as mean values ± SD for ten independent assays. To determine stability of CDC (**B** and **D**), two ch14.18/CHO concentrations 1.0 µg/ml (closed circles) and 0.1 µg/ml (open circles) prepared in 12.5% serum of a healthy donor were analyzed with established cytotoxicity assay. Samples were subjected to either storage at room temperature for up to 96 h (**B**) or to five freeze-thaw cycles (**D**). Rituximab containing (closed triangles) and Ab-free controls (open triangles) were included as described in Materials and Methods. Data are shown as mean CDC values ± SE of two independent experiments performed at least in triplicates.

The within-assay precision and the inter-assay variation were assessed according to international consensus recommendations for the bioanalytical method validation [18]. We defined values of CV for both within- and inter-assay variation up to 20% as acceptable. For within-assay precision, CDC of ten samples containing 1.0 µg/ml ch14.18/CHO prepared in serum of a healthy donor (D1) was performed and CDC data were used for calculation of CV (Fig. 8A). Each sample was analyzed in duplicates on the same plate. Analysis revealed a CV of 9% indicating reproducible performance of the assay. The inter-assay CV was determined in triplicates by changing the operators on ten different days. This analysis revealed a CV of 12% (Fig. 8C) clearly showing that results obtained are operator-independent and consistent over time.

To accept or reject experimental runs, QCs containing a known concentration of ch14.18/CHO (1.0 µg/ml) in serum of a healthy donor (D1) were included in each analysis. The deviation coefficients were calculated using QC results obtained after each analysis and the respective QC nominal value. The differences did not exceed 20% allowing analysis acceptance.

In order to test sample stability and subsequent use in the bioassays described after distinct storage conditions, we used healthy donor serum (D1) containing two defined concentrations of ch14.18/CHO (1.0 and 0.1 µg/ml). Neither storage at RT for up to 96 hours (Fig. 8B) nor up to three repeated freeze thaw cycles (Fig. 8D) revealed significant changes in the level of CDC-mediated by ch14.18/CHO. Next, we analyzed leukocyte count and viability in blood samples using two anticoagulants, EDTA and sodium-heparin, respectively (Table 2). In general, sodium-heparin anticoagulated whole blood revealed longer stability compared to EDTA anti-coagulated blood with acceptable quality for up to 48 h storage at RT. Blood stored at RT for 72 h negatively affected both leukocyte count and viability independent on anticoagulants. These data were confirmed by ADCC analysis using effector cells prepared from samples stored as described above.

**Table 2:** Effect of anticoagulants on leukocyte count and viability.

Leukocyte viability (%)				
storing time (h)	0	24	48	72
EDTA-blood	98.5±0.5	88.5±3.5	80.0±6.0	80. 0±6.0
Na-heparin-blood	99.0±0.0	96.5±2.5	95.5±2.5	91.0±5.0
**Leukocyte count (1×10^9^ cells/l)**				
storing time (h)	0	24	48	72
EDTA-blood	2.00±0.00	1.00±0.22	0.71±0.29	0.53±0.21
Na-heparin-blood	2.00±0.00	1.84±0.08	1.68±0.43	1.33±0.53

Leukocytes were isolated from sodium-heparin- and EDTA whole blood samples followed by analysis of cell viability and count. Values are represented as mean ± SE of two independent experiments.

We also evaluated the impact of storage conditions on the granulocyte rich effector cell fractions. We prepared them from blood samples after 2 and 4 hours of storing at RT and included granulocyte rich fractions freshly isolated, but cultured for 64 h as described in Materials and Methods (data not shown). We could observe a reduction in granulocyte count by only 17% and 21% at 2 and 4 hour time points, respectively. Interestingly, the cell viability of granulocytes rich fractions was 99% at both time points. The cell count of granulocytes was reduced by 60% after 64 h of culture with nearly 100% viability consistent with the known fragility of this cell population. This finding is in line with a reduction of the cytotoxic efficacy by 50% compared to fresh isolated granulocytes at the 64 h time point. Therefore, analysis of granulocyte mediated ADCC clearly requires fresh blood samples and timely processing.

In summary, sodium-heparin anti-coagulated blood and serum can be stored together at RT for up to 48 h allowing for a timely transfer of the samples from clinical study sites for central lab analysis of CDC and ADCC using the described bioassays.

### Evaluation of CDC and ADCC bioassays in neuroblastoma patients

Serum samples and anti-coagulated blood (heparin) were collected from three selected NB patients (patient 1, patient 2, patient 3) treated within a Phase I/II clinical trial where patients are treated with a cumulative dose of ch14.18/CHO (100 mg/m^2^/cycle) infused over a time period of ten days (Eudra CT: 2009-018077-3). The collected samples were taken at base line before start of immunotherapy and on day 8 after start of antibody infusion. The collected serum samples were used after heat-inactivation for ADCC- and untreated for CDC-assays containing a patient-specific level of ch14.18/CHO Ab as a result from the treatment (i.e. without exogenous addition of ch14.18/CHO). The serum levels of ch14.18/CHO were determined as previously described [11] (P1: 13.30±0.08 µg/ml, P2: 15.14±0.24 µg/ml, P3: 11.39±0.24 µg/ml).

The effector cells were also used without further *ex vivo* stimulation at an E:T ratio of 40∶1.

**Figure 9:**
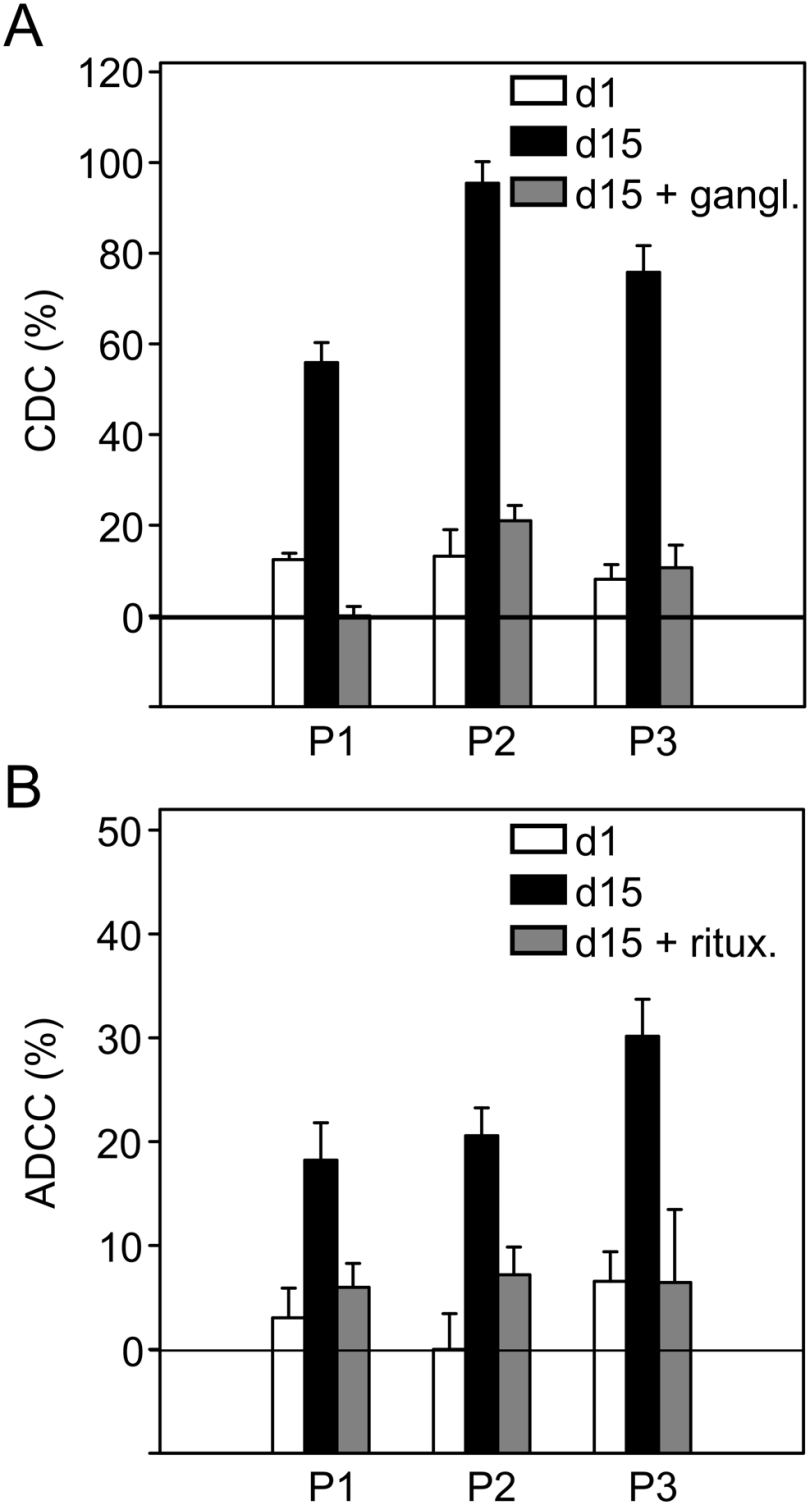
Evaluation of CDC and ADCC in high-risk NB patients treated with a combination of IL-2 and ch14.18/CHO mAb. Serum samples (12.5% final concentration) and effector cells (E:T ratio of 40∶1) collected from three selected NB patients (patient 1, patient 2, patient 3) treated with 100 mg/m^2^ ch14.18/CHO in combination with 6×10^6^ IU/m^2 ^s.c. IL-2 were analyzed for CDC (**A**) and ADCC (**B**) on two days (day 1, prior to Ab infusion, white column and day 15, eight days after the start of Ab application, black column) using both established bioassays. Patient-specific CDC and ADCC were calculated as described in Materials and Methods. Rituximab and anti-Id Ab ganglidiomab containing controls (grey columns) were included as described in Materials and Methods. Data are shown as mean values ± SE of independent experiments performed at least in triplicates. *t*-test; *P<0.05.

ADCC and CDC analyzed with these blood and serum samples resulted in a significant increase of CDC (Fig. 9A) and ADCC (Fig. 9B) in all treated patients. When serum samples were replaced with rituximab or pre-incubated with excess of anti-Id Ab ganglidiomab, only background cytotoxicity was observed in both bioassays. In summary, the ADCC and CDC methodology described here indicate GD_2_-specific cytotoxic activity in blood and serum samples obtained from patients treated with ch14.18.

## Discussion

Ab are important therapeutics for the treatment of malignant disease and many mechanisms have been proposed to explain the clinical anti-tumor activity of mAb targeting a tumor associated antigen. Some Ab act through their ability to disrupt signaling pathways involved in the maintenance of their malignant phenotype such as blockade of ligands binding to growth factor receptors (e.g.cetuximab; EGFR) [22]. However, the ability of Ab to initiate a tumor-specific immune response has been less well recognized. Several studies have established the importance of ADCC-mediated through Fc-Fcγ receptor interaction as well as CDC for the *in vivo* anti-tumor effects of mAb. This was demonstrated in mice which lack Fcγ receptor expression or which are deficient in C1q, a critical component of the complement system. In both types of mouse strains the anti-tumoral activity of anti-CD20 Ab was diminished compared to wild-type strains [23], [24].

The initiation of a tumor-specific immune response is also an important mechanism of action related to passive immunotherapies based on application of anti-GD_2_ mAb. This intervention emerges as an established concept for the treatment of NB, a challenging malignancy in pediatric oncology [25]. The target of this strategy is ganglioside GD_2_, a glycolipid expressed on the cell surface of malignant cells of neuroectodermal origin devoid of an intracellular signaling domain. Therefore, the evaluation of tumor-specific anti-NB immune responses mediated by ADCC and CDC should capture the core components of the clinical activity observed with this class of anti-tumor Ab.

Here, we describe two rapid non-radioactive and non-toxic ADCC and CDC assays allowing for analysis of samples collected from NB patients treated with anti-GD_2_ Ab ch14.18/CHO for the purpose of immune monitoring of ongoing clinical trials. Importantly, we demonstrated increased GD_2_ specific CDC and ADCC in three selected high-risk NB patients treated with ch14.18/CHO indicating expected effector functions of ch14.18/CHO in treated patients.

Among non-radioactive agents that can be used for such cytotoxicity assays, calcein-AM is one of the most prominent fluorescent dyes [26] used for labeling of target cells. Here, expression of multidrug transporter P-glycoprotein (MDR-1) is a hindering factor extruding calcein from target cells independently of CDC or ADCC. To avoid this problem, we selected LA-N-1 cells from a panel of NB cell lines. LA-N-1 showed low P-glycoprotein expression levels and subsequently low background signals after calcein labeling. Moreover, the most homogeneous and strongest GD_2_ expression and the highest level of GD_2_-specific lysis in contrast to all other NB cell lines of the available panel resulted in the decision to establish CDC and ADCC bioassays with LA-N-1 target cells. We also aimed to optimize the bioassays to reduce both preparation time required for effector cell isolation as well as sample volume, which is particularly important in a pediatric population. To this end, a minimum of 100 µl of serum and a blood sample containing 200,000 leukocytes are sufficient which can be isolated within 10 min from small blood volume of less than 500 µl.

A second challenge to overcome is the fact that European NB trials are generally multi-center in nature and require sample transfer to a central laboratory for analysis. Therefore, the impact of shipping conditions is of great importance. According to our present data, serum samples could be stored at RT for four days and subjected three times to repeated thawing and freezing without significant influence on CDC. For the analysis of ADCC, blood needs to be anticoagulated. Although EDTA is the most commonly used anticoagulant, it is known that it affects both erythrocytes and leukocytes, causing membrane damage [27]. Here, we compared the impact of two anti-coagulants, EDTA and sodium-heparin, on leukocyte count and viability and count in blood samples stored at RT for 72 h. We could not observe any significant changes in cell-viability and -count of effector cells in sodium-heparin-anticoagulated blood for up to 48 h at RT in contrast to EDTA-anticoagulated blood samples. In contrast, the analysis of granulocyte rich effector cell fractions revealed a reduction of granulocyte counts and anti-tumor cytotoxic activity during storage of blood samples. Therefore, the evaluation of granulocyte-mediated ADCC as a biomarker requires timely processing and analysis, which is unsuitable for central lab testing in a multicenter setup of a clinical trial. In summary, serum and in sodium-heparin-anticoagulated blood samples can be shipped within 48 h for central laboratory bioassay analysis. We analyzed CDC and ADCC in three selected high-risk NB patients participating in this trial and could observe a significant increase of both anti-tumor CDC and ADCC after Ab administration indicating beneficial effects of this immunotherapy.

In order to permit a valid interpretation of data from functional assays employed for quantitative determination of cytotoxicity in biological samples, reproducible and reliable data must be generated operator independent in a laboratory performing such analyses [28]. To validate the established assay and to show its reproducibility, accuracy and precision, both within-assay and inter-assay precision analysis were determined and QC were included in each analytical run according to the international guidelines for bioanalytical method validation [18]. We found CV of both within-assay and inter-assay to be under 20% indicating operator-independent and reproducible performance over time.

Recently, a concept of a personalized medicine in NB treatment becomes more important [29]. In line with the fact that the extraordinary diversity of the clinical course in NB patients is reflected by a substantial genetic variation [29], we observed inter-individual differences in a basic Ab-independent cytotoxicity in NB patients as well as about 40% inter-individual differences in healthy donors anylzed. By using the bioassays described here in combination with pharmacokinetic [11] and human anti-chimeric response analysis [12], we aim to monitor patients currently treated in clinical trials with ch14.18/CHO in order to work towards the establishment of a set of biomarkers potentially predictive of response.

Taken together, we reported two validated non-toxic and non-radioactive *in vitro* assays for rapid analysis of patient-specific CDC and ADCC in small volume samples collected from NB patients treated with anti-GD_2_ Ab. These two assays allow reliable analysis within 48 h after sample collection for standard laboratory purposes and large-scale applications in clinical trials and are therefore a suitable tool for immune monitoring also in the multicenter setting.

## References

[1] Modak S, Cheung NK (2007) Disialoganglioside directed immunotherapy of neuroblastoma. Cancer Invest 25: 67–77. 17364560 10.1080/07357900601130763

[2] Yang RK, Sondel PM (2010) Anti-GD_2_ Strategy in the Treatment of Neuroblastoma. Drugs Future 35: 665. 21037966 10.1358/dof.2010.035.08.1513490 PMC2964668

[3] Shuptrine CW, Surana R, Weiner LM (2012) Monoclonal antibodies for the treatment of cancer. Semin Cancer Biol 22: 3–13. 22245472 10.1016/j.semcancer.2011.12.009 PMC3288558

[4] Saito M, Yu RK, Cheung NK (1985) Ganglioside GD_2_ specificity of monoclonal antibodies to human neuroblastoma cell. Biochem Biophys Res Commun 127: 1–7. 2579648 10.1016/s0006-291x(85)80117-0

[5] Cheresh DA, Rosenberg J, Mujoo K, Hirschowitz L, Reisfeld RA (1986) Biosynthesis and expression of the disialoganglioside GD_2_, a relevant target antigen on small cell lung carcinoma for monoclonal antibody-mediated cytolysis. Cancer Res 46: 5112–5118. 3019521

[6] Simon T, Hero B, Faldum A, Handgretinger R, Schrappe M, et al (2004) Consolidation treatment with chimeric anti-GD_2_-antibody ch14.18 in children older than 1 year with metastatic neuroblastoma. J Clin Oncol 22: 3549–3557. 15337804 10.1200/JCO.2004.08.143

[7] Simon T, Hero B, Faldum A, Handgretinger R, Schrappe M, et al (2011) Long term outcome of high-risk neuroblastoma patients after immunotherapy with antibody ch14.18 or oral metronomic chemotherapy. BMC Cancer 11: 21. 21244693 10.1186/1471-2407-11-21 PMC3031264

[8] Yu AL, Gilman AL, Ozkaynak MF, London WB, Kreissman SG, et al (2010) Anti-GD_2_ antibody with GM-CSF, interleukin-2, and isotretinoin for neuroblastoma. N Engl J Med 363: 1324–1334. 20879881 10.1056/NEJMoa0911123 PMC3086629

[9] Zeng Y, Fest S, Kunert R, Katinger H, Pistoia V, et al (2005) Anti-neuroblastoma effect of ch14.18 antibody produced in CHO cells is mediated by NK-cells in mice. Mol Immunol 42: 1311–1319. 15950727 10.1016/j.molimm.2004.12.018

[10] Ladenstein R, Weixler S, Baykan B, Bleeke M, Kunert R, et al (2013) Ch14.18 antibody produced in CHO cells in relapsed or refractory Stage 4 neuroblastoma patients: a SIOPEN Phase 1 study. MAbs 5: 801–809. 23924804 10.4161/mabs.25215 PMC3851232

[11] Siebert N, Seidel D, Eger C, Brackrock D, Reker D, et al (2013) Validated detection of anti-GD_2_ antibody ch14.18/CHO in serum of neuroblastoma patients using anti-idiotype antibody ganglidiomab. J Immunol Methods 398–399: 51–59. 10.1016/j.jim.2013.09.008 24055592

[12] Siebert N, Eger C, Seidel D, Juttner M, Lode HN (2014) Validated detection of human anti-chimeric immune responses in serum of neuroblastoma patients treated with ch14.18/CHO. J Immunol Methods 407: 108–115. 24727144 10.1016/j.jim.2014.04.001

[13] Seeger RC, Rayner SA, Banerjee A, Chung H, Laug WE, et al (1977) Morphology, growth, chromosomal pattern and fibrinolytic activity of two new human neuroblastoma cell lines. Cancer Res 37: 1364–1371. 856461

[14] Bumol TF, Reisfeld RA (1982) Unique glycoprotein-proteoglycan complex defined by monoclonal antibody on human melanoma cells. Proc Natl Acad Sci U S A 79: 1245–1249. 6175965 10.1073/pnas.79.4.1245 PMC345938

[15] Keshelava N, Seeger RC, Groshen S, Reynolds CP (1998) Drug resistance patterns of human neuroblastoma cell lines derived from patients at different phases of therapy. Cancer Res 58: 5396–5405. 9850071

[16] Biedler JL, Helson L, Spengler BA (1973) Morphology and growth, tumorigenicity, and cytogenetics of human neuroblastoma cells in continuous culture. Cancer Res 33: 2643–2652. 4748425

[17] Lode HN, Schmidt M, Seidel D, Huebener N, Brackrock D, et al (2013) Vaccination with anti-idiotype antibody ganglidiomab mediates a GD_2_-specific anti-neuroblastoma immune response. Cancer Immunol Immunother 62: 999–1010. 23591980 10.1007/s00262-013-1413-y PMC11028789

[18] DeSilva B, Smith W, Weiner R, Kelley M, Smolec J, et al (2003) Recommendations for the bioanalytical method validation of ligand-binding assays to support pharmacokinetic assessments of macromolecules. Pharm 20: 1885–1900. 10.1023/b:pham.0000003390.51761.3d 14661937

[19] Homolya L, Hollo Z, Germann UA, Pastan I, Gottesman MM, et al (1993) Fluorescent cellular indicators are extruded by the multidrug resistance protein. J Biol Chem 268: 21493–21496. 8104940

[20] Gelderman KA, Tomlinson S, Ross GD, Gorter A (2004) Complement function in mAb-mediated cancer immunotherapy. Trends Immunol 25: 15164. 10.1016/j.it.2004.01.008 15036044

[21] Sorkin LS, Otto M, Baldwin WM III, Vail E, Gillies SD, et al (2010) Anti-GD_2_ with an FC point mutation reduces complement fixation and decreases antibody-induced allodynia. Pain 149: 135–42. 20171010 10.1016/j.pain.2010.01.024 PMC3755890

[22] Li S, Schmitz KR, Jeffrey PD, Wiltzius JJ, Kussie P, et al (2005) Structural basis for inhibition of the epidermal growth factor receptor by cetuximab. Cancer Cell 7: 301–311. 15837620 10.1016/j.ccr.2005.03.003

[23] Clynes RA, Towers TL, Presta LG, Ravetch JV (2000) Inhibitory Fc receptors modulate in vivo cytotoxicity against tumor targets. Nat Med 6: 443–446. 10742152 10.1038/74704

[24] Di GN, Cittera E, Nota R, Vecchi A, Grieco V, et al (2003) Complement activation determines the therapeutic activity of rituximab in vivo. J Immunol 171: 1581–1587. 12874252 10.4049/jimmunol.171.3.1581

[25] Ahmed M, Cheung NK (2014) Engineering anti-GD_2_ monoclonal antibodies for cancer immunotherapy. FEBS Lett 588: 288–297. 24295643 10.1016/j.febslet.2013.11.030

[26] Lichtenfels R, Biddison WE, Schulz H, Vogt AB, Martin R (1994) CARE-LASS (calcein-release-assay), an improved fluorescence-based test system to measure cytotoxic T lymphocyte activity. J Immunol Methods 172: 227–239. 7518485 10.1016/0022-1759(94)90110-4

[27] Lewis SM, Stoddart CT (1971) Effects of anticoagulants and containers (glass and plastic) on the blood count. Lab Pract 10: 787–792. 4999452

[28] Smolec J, DeSilva B, Smith W, Weiner R, Kelly M, et al (2005) Bioanalytical method validation for macromolecules in support of pharmacokinetic studies. Pharm Res 9: 1425–1431. 10.1007/s11095-005-5917-9 16132353

[29] Tonini GP, Nakagawara A, Berthold F (2012) Towards a turning point of neuroblastoma therapy. Cancer Lett 326: 128–134. 22922304 10.1016/j.canlet.2012.08.017

